# Investigation of an Effective Anchoring Length of CFRP Tapes Used to Strengthen Steel Thin-Walled Beams with a Rectangular Cross-Section Subjected to Four-Point Bending

**DOI:** 10.3390/ma16072907

**Published:** 2023-04-06

**Authors:** Ilona Szewczak, Malgorzata Snela, Patryk Rozylo

**Affiliations:** 1 Faculty of Civil Engineering, Lublin University of Technology, 40 Nadbystrzycka Str., 20-618 Lublin, Poland; 2 Faculty of Mechanical Engineering, Lublin University of Technology, Nadbystrzycka 36, 20-618 Lublin, Poland

**Keywords:** thin-walled structures, experimental study, FEM analysis, effective anchoring

## Abstract

In order to design an optimal reinforcement of steel thin-walled beams with composite materials, it is worth analyzing two important, although often overlooked issues, which are the selection of the appropriate thickness of the adhesive layer and the effective anchoring length of the composite tape. This paper, which is part of a wider laboratory study devoted to the strengthening of thin-walled steel profiles, focuses on the second issue. The paper involves a description of laboratory four-point bending tests during which ten thin-walled steel beams made of a rectangular section with dimensions of 120 × 60 × 3 and a length of 3 m were tested. Two beams were taken as reference beams, and the other eight were reinforced using Sika CarboDur S512 carbon fiber composite tape, assuming four different effective anchorage lengths. The impact of the length of the anchoring of the composite tape on the value of the displacements and strains of the tested beams and on the value of the destructive load that caused tape detachment was analyzed. The following phase was numerical analyses carried out in the Abaqus program, which showed high consistency with the results of laboratory tests. In reference to the conducted tests, it was observed that the increase in the anchoring length of the composite tape has a slight impact on the change in the value of strains and displacements in the tested beams. Nevertheless, the increase in the effective anchorage length has a significant impact on the load value at which the composite tapes are detached from the surface of the steel thin-walled beam.

## 1. Introduction

The intensive increase in the use of thin-walled steel structures in construction makes it necessary to find innovative and non-invasive methods of strengthening such elements. One such method may be reinforcing steel structures with glued composite tapes, which is successfully used in the case of reinforced concrete, reinforced concrete structures or steel structures made of hot-rolled elements. It should be noted that when designing structural reinforcements using composite tapes, it is important to adopt the right thickness of the adhesive layer, the correct length of the anchoring of composite tapes and even the correct shape of the glue. As there are no clear guidelines and recommendations in the documents made available by manufacturers regarding the reinforcement of specific construction materials (steel, concrete, reinforced concrete, wood and masonry elements), it is important to perform a number of laboratory tests that allow for the formulation of clear design recommendations.

The problem of selecting the correct adhesive layer thickness in relation to thin-walled structures has been touched upon by the authors in [1,2]. The conducted tests showed a significant impact of the thickness of the bonded joint layer on the moment of tape detachment at the steel–glue contact along with the increase in the thickness of the adhesive layer; the load value at which the tape detachment occurred decreased. The problem of selecting the correct shape of the end of the glaze in the case of strengthening steel structures has been described in papers [3,4].

**Figure 1:**
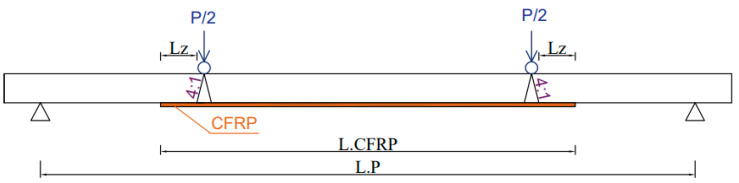
The anchorage length Lz.

In the case of studies devoted to the correct anchoring length of composite tapes, a number of studies regarding the reinforcement of concrete or reinforced concrete structures [5,6,7,8,9] and few studies devoted to the study of the optimal anchoring length of composite tapes in bridge [10] or hot-rolled steel structures [11,12,13,14] have been found. The following conclusions appeared in the above-mentioned literature items: the authors of the paper [12] showed that nearly 98% of the load value is transferred by the composite tape to the steel element on the last 10 cm of the tape length, which allows for the conclusion that 100 mm can be assumed as the effective length of the tape anchoring; in the paper [13], it was found that on the basis of the CHS (Circular Hollow Section) steel cross-section tests, the effective anchoring length can be assumed as 75 mm, while in the paper [10] on the basis of the study of steel bending elements, the authors assumed an effective anchoring length of 203 mm. In the paper [10], the authors defined the effective anchoring length of composite tapes as the minimum length that allows the transfer of the maximum load by composite tapes. According to this definition, a diagram (Figure 1) appeared in the work [14], which was also used in this work to determine the anchorage length (Lz). In Figure 1, L_CFRP_ is the total length of the composite tape, and L_P_ is the span in the axes of the supports.

The author of the works [3,14], based on the examination of the bent INP 140 beams lengths of 190 cm, stated that the transfer of almost the entire value of the load between the tape and the beam takes place on the last 70 mm of the tape length; however, only in the case of using an effective anchorage length of 265 mm, he obtained yielding of the tested beams before the detachment of the composite tapes. The author of the work [15] stated that the effective anchorage length depends on the FRP (Fiber-reinforced Plastic) modulus and the equivalent deformation of adhesion; it increases with the increase of the above parameters. At the same time, the effective anchorage length decreases with increasing adhesive tensile strength. The analyses described in the paper [15] are very valuable, but they do not provide simple implementation recommendations.

The studies described above prompted the authors to determine the correct anchoring length of CFRP (carbon fiber reinforcement polymer) tapes in tested steel thin-walled structures of the sigma type [16]. Based on the test performed within the four-point bending scheme, it was found that as the anchoring length increases, the value of strains in the tested beams decreases negligibly; therefore, from an economic point of view, the most favorable tape anchoring length is 70 mm. Due to the complex work of the monosymmetric beam (beam subjected to bending and torsion) and thus the possibility of measurement errors, it was decided to conduct another series of tests on bent beams with a thin-walled rectangular cross-section and develop an advanced numerical model in the Abaqus program in order to formulate conclusions regarding the selection of the right anchoring length of composite tapes which are used to reinforce steel thin-walled elements. The subject of testing on engineering structures with hollow sections in terms of reinforcing them with composite materials is the subject of testing and analyses by many researchers [17,18].

## 2. Laboratory Tests

The laboratory tests included ten thin-walled steel beams made of S235-grade steel. Beams with a length of 3 m and dimensions of 120 × 60 mm and a wall thickness of 3 mm were subjected to an endurance test in a four-point bending scheme. The two beams were reference samples and were not reinforced with composite tapes. The remaining beams were reinforced by gluing composite tapes with different anchoring lengths to them. Composite tapes were placed on the lower flange of the beams subjected to testing. The symbols of the individual samples and the length of the tapes are listed in Table 1. The anchorage length of the tape was defined on the basis of Figure 1 (Lz). The effective anchorage length is the shortest anchorage length that maximizes the load transferred to the CFRP tape.

The beams were reinforced with CFRP tapes—Sika CarboDur S512 composite tapes—with cross-sectional dimensions of 50 × 1.2 mm, which were connected to the surface of the beams with SikaDur-30 adhesive.

The thickness of the adhesive layer used in the study was 1.3 mm. The basic strength parameters of the tapes (determined on the basis of material tests) and the adhesive (obtained from the manufacturer’s material sheets) are summarized in Table 2.

**Figure 2:**
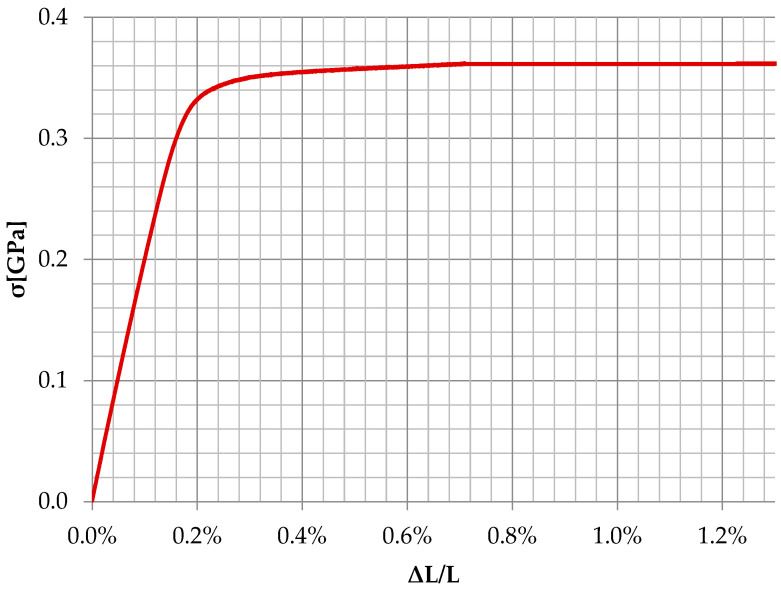
Material characteristics of steel.

On the other hand, the strength parameters of the steel from which the thin-walled beams were made were determined on the basis of testing five samples cut from profiles (Figure 2). On this basis, Young’s modulus of 207.87 GPa and Poisson’s coefficient of 0.307 were adopted.

**Figure 3:**
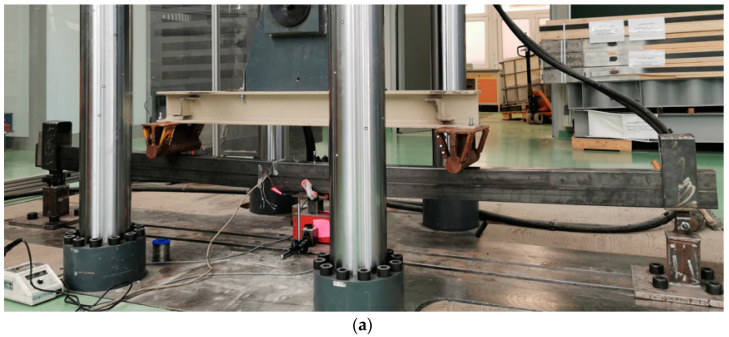

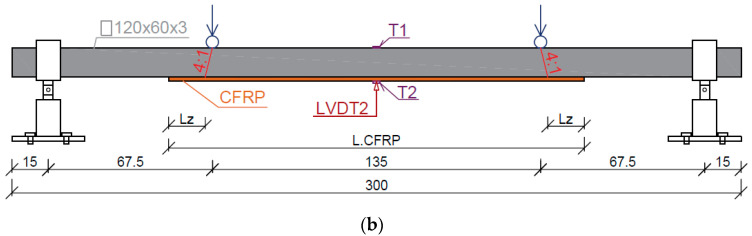
Laboratory stand: (**a**) photo, (**b**) scheme.

Thin-walled steel beams were matted on the surface of the lower flanges and degreased, and CFRP tapes were glued. The beams were stored under laboratory conditions and tested after 28 days in a ZwickRoell (ZwickRoell GmbH & Co. KG, Ulm, Germany) testing machine in a four-point bending scheme. This time (28 days) was required in furtherance of achieving the full strength of the bonded joint. During the tests, the load increase was obtained by the movement of the press piston. The piston speed was assumed to be 1 mm/min, and the force value was measured in intervals of 0.01 s. In the entire cycle of laboratory tests, the deflection of the tested beams was measured using the LVDT2 inductive sensor located in the middle of the beam span, and the strain using two electrofusion strain gauges (with detailed parameters—TENMEX TFs-10 120 Ω ± 0.2%—marked in Figure 3 as T1 and T2) located in the middle of the upper and lower flanges in the middle of the beam span. The photo of the test stand and the scheme of the laboratory stand are presented in Figure 3.

## 3. Laboratory Tests Results

**Figure 4:**
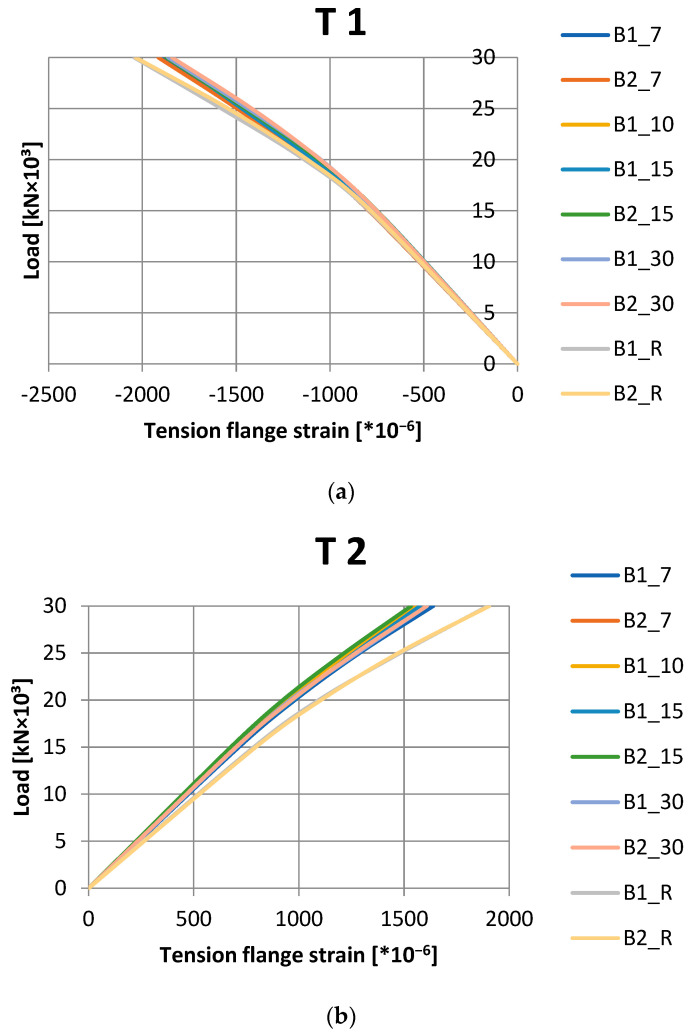

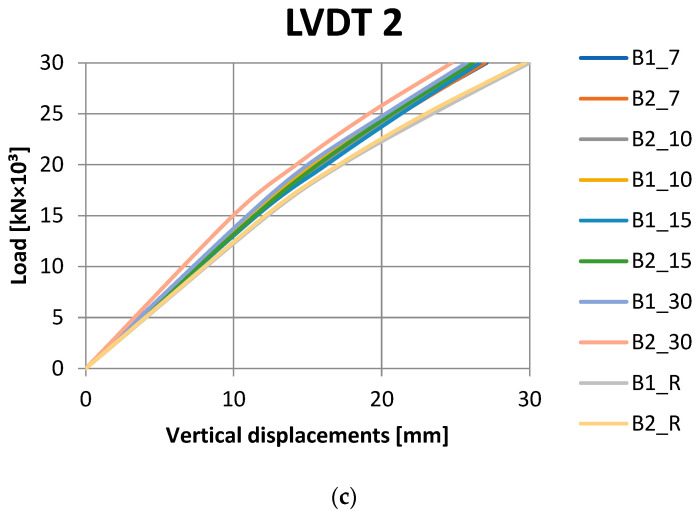
The results of laboratory tests: (**a**) strain in upper flange, (**b**) strain in lower flange, (**c**) vertical displacements.

In laboratory tests, an LVDT2 inductive sensor was used to measure the vertical deflection of the beams, whereas strains in the compression and tension flanges were measured using T1 and T2 electrofusion strain gauges. The results are presented in the graphs in Figure 4. The presentation of the results was limited to a load of 30 kN because, at a load value of 30.4 kN, tape detachment was observed in one of the tested beams.

In the case of the measurement of both strain and displacements, the obtained results indicate that the application of CFRP tapes enables a significant limitation of these parameters. As the anchoring length of the CFRP tapes increases, the displacement and strain values in the compression flange decrease. Due to the low readability of the diagrams presented in Figure 4, Table 3 is prepared with the results of strain and displacements for the load level of 30 kN presented.

The use of CFRP tapes with a minimum anchorage length allows the strain in the upper compression flange to be reduced by 6.3%, and the vertical deflections in the middle of the beam span to be reduced by 9.3%. On the other hand, the application of the maximum analyzed anchorage length reduces these parameters by 9.1 and 15.4%, respectively. In the case of strain values in the lower flange, the read results do not show a clear relationship; the greatest limitation of strain was obtained for the anchoring length of 15 cm. With an anchorage length of 7 cm, a reduction in deformation of 16.5% was obtained, and in the case of 15 cm, it was 18.3%.

Based on the conducted tests, it can be concluded that the change in the anchorage length has a negligible impact on the change in the strain value (approx. 3%). When measuring deflections, the effect of the tape length is also minor and amounts to about 6%.

## 4. Numerical Simulations

Numerical simulations were performed using the Abaqus^®^ software (2022, Dassault Systemes Simulia Corporation, Velizy Villacoublay, France). In the case of numerical simulations, the use of the finite element method (FEM) allowed for the development of five numerical models representing experimental research conducted in parallel. The first model represented a numerical model where there was a steel beam unreinforced with composite tapes, which was supported on supports and loaded with a loading component. The other four numerical models constituted a modification of the basic model, where each model used a reinforcement of the steel reference beam through the use of composite strips with different lengths, which were directly bonded to the beam (to the underside of the steel profile). The aforementioned models differed only in the length of the composite strips used, where the lengths were, respectively, 155, 161, 171 and 201 cm. During the FEA analysis, it was possible to compare the effect of the length of the bonded composite tapes, which was used to reinforce the profile of the steel beam and relate the results to the unreinforced beam.

**Figure 5:**
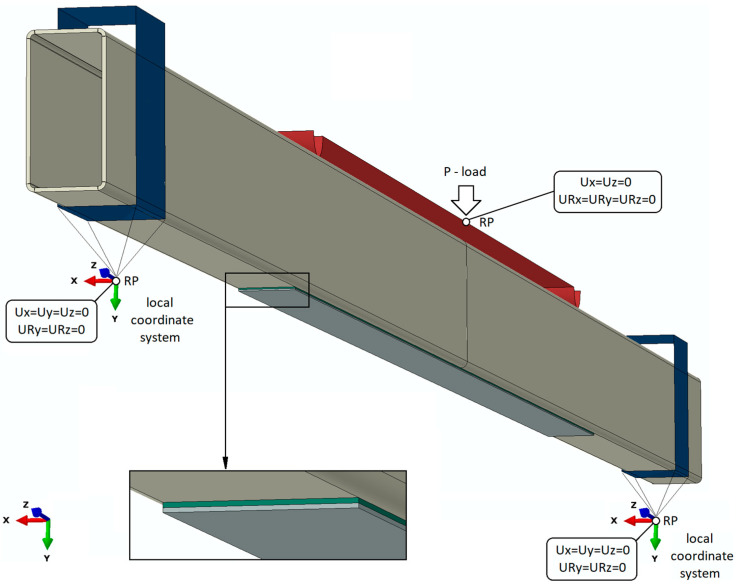
Boundary condition of FEM model.

The boundary conditions were reflected adequately for actual research. The boundary conditions were defined using contact interactions between the steel beam and the supports and the loading element (where contact in the normal and tangential directions was considered with a friction coefficient of 0.2). Moreover, in the case of models reinforced with composite tape, the composite tape was connected to the adhering layer, while the adhesive was connected to the underside of the steel profile, using a Tie interaction. Within the elements representing the loading element and the supports, special reference points were defined, where the necessary degrees of freedom were defined, as shown in Figure 5.

**Figure 6:**
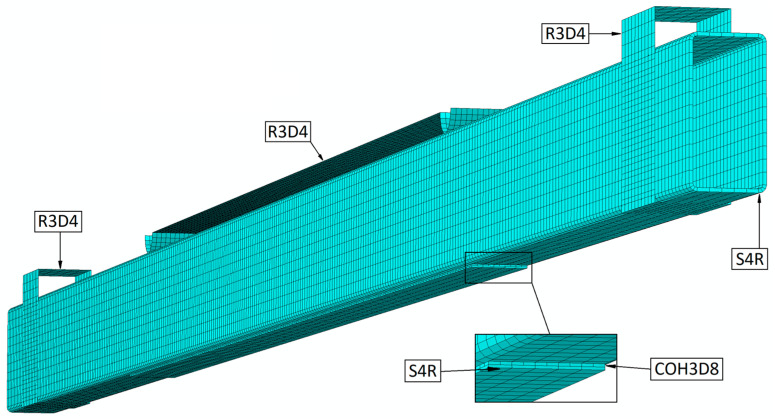
Discrete FEM model.

Discrete models were prepared based on the use of several types of finite elements, thus enabling the developed numerical models to be correctly reflected. The steel beam was prepared using S4R-type finite elements (shell elements with a linear shape function and four nodes each). The supports and the loading element were prepared using non-deformable finite elements of R3D4. The composite tape was modeled using shell elements (S4R-type elements), similar to the steel beam. The adhesive layer was prepared using cohesive elements of a COH3D8-type (elements with eight nodes each). The use of the above-mentioned different types of finite elements for the modeled components made it possible, among other things, to simulate the bending phenomenon of a steel beam, taking into account the failure of the adhesive bond (cohesive layer) in the case of models reinforced with different lengths of composite tapes. Using this approach, it was possible to simulate damage within the adhesive layer when its limiting parameters were exceeded. The basic (reference) form of the discrete model, without the adhesive bond (with composite tapes), was composed of 16,900 finite elements as well as 17,325 computational nodes. The other discrete models considered a different number of finite elements and computational nodes, where, respectively, the model with a 155 cm tape had 20,775 finite elements and 25,103 nodes, the model with a 161 cm tape had 20,925 finite elements and 25,403 nodes, the model with a 171 cm tape had 21,175 finite elements and 25,903 nodes, and the model with a 201 cm tape had 21,925 finite elements and 27,403 nodes. The steel beam, as well as the composite tape, were described by finite elements with dimensions of approximately 10 × 10 mm, while the adhesive layer was described by numerical analysis with elements of about 5 × 5 mm. The numerical simulations carried out investigated the effect of mesh density on the quality of the test results, but no significant discrepancies were observed either in a quantitative or qualitative context. Relevant information on similar studies is presented in the paper [19]. The discrete model is shown in Figure 6 below (model with a tape length of 155 cm).

The material parameters of a steel beam made of a rectangular section profile were described using an elastic–plastic material model (material parameters are presented earlier at the stage of the description of the subject of research). The material data used in the analysis, both for the composite tape and the adhesive, were taken from [1,20].

## 5. Results of Numerical Analyses

**Figure 7:**
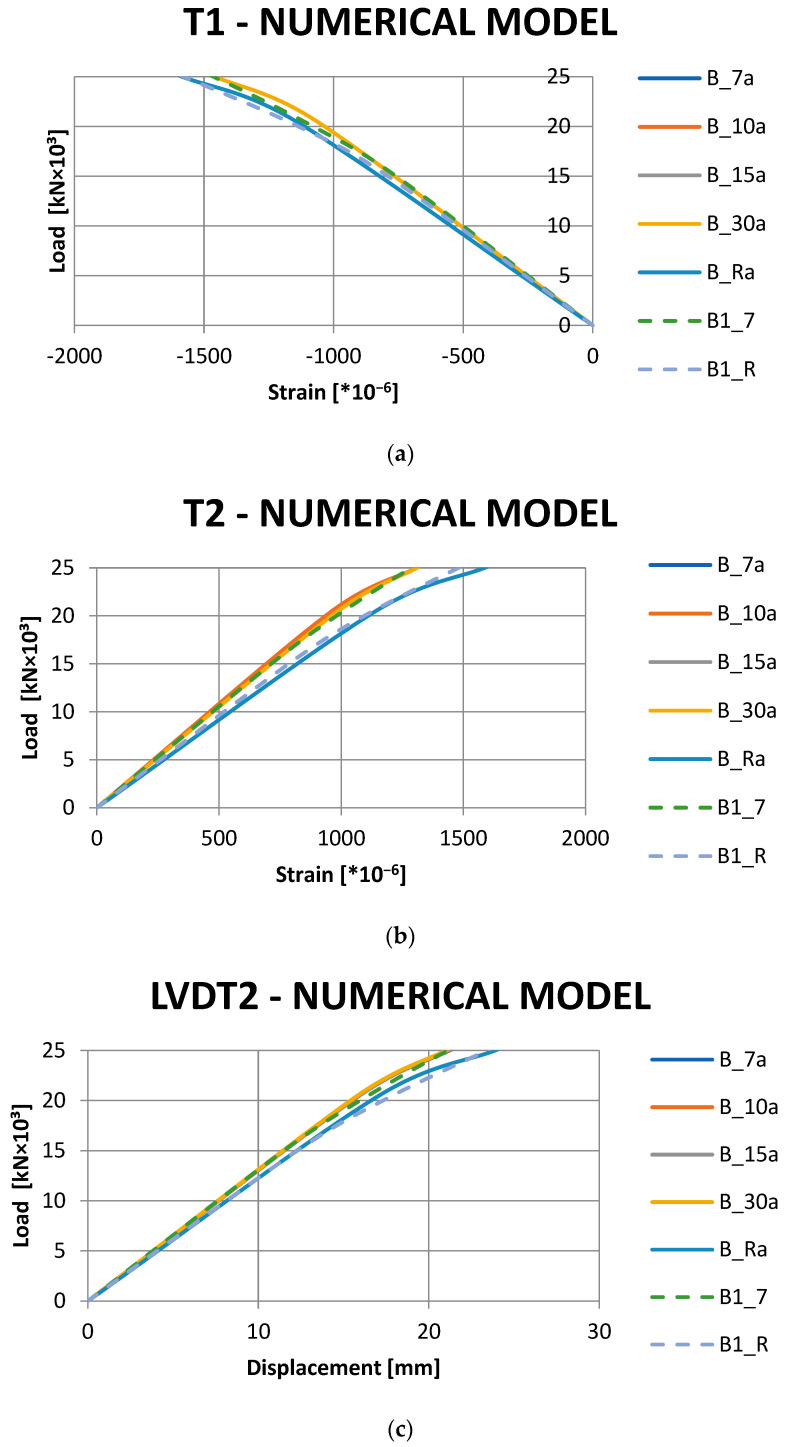
Results of numerical analyses: (**a**) strain values at the T1 strain gauge location, (**b**) strain values at the T2 strain gauge location, (**c**) displacement values at the LVDT2 sensor measurement location.

**Figure 8:**
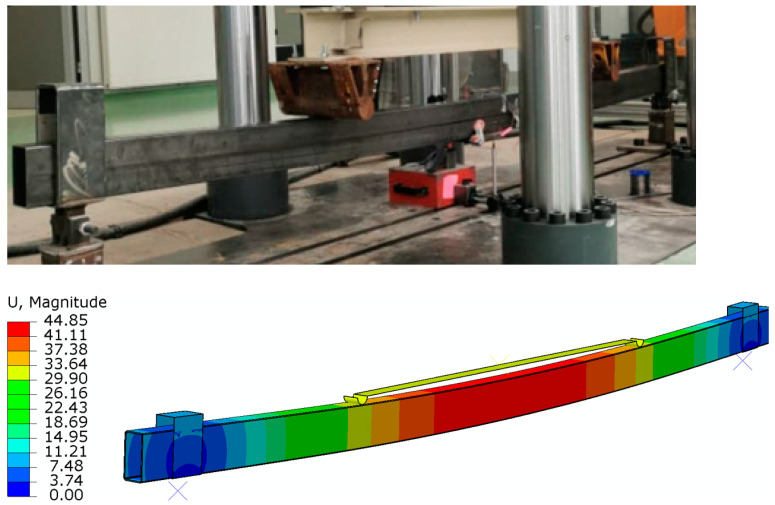
The beam failure obtained in laboratory tests and numerical analyses.

During numerical analyses, a number of numerical models were developed, differing in the length of the CFRP tape. The individual numerical models were named in relation to the corresponding beams tested in the laboratory, adding the letter ‘a’ at the end: B_7a, B_10a, B_15a, B_30a and B_Ra. The detailed results of laboratory tests carried out were highly consistent with the results obtained from numerical analyses, as shown in Figure 7, adding for comparison the results obtained in laboratory tests by the reference beam B1_R and the reinforced beam B1_7. Furthermore, the behavior of the beam during laboratory tests is consistent with that obtained from numerical analyses (Figure 8).

As shown in Figure 7, the results of the numerical analyses and laboratory tests show the same trend. In the numerical analyses, the addition of reinforcement in the form of CFRP tapes allowed strain to be reduced at points T1 and T2 in relation to the beam without reinforcement by 8 and 17%, respectively, and vertical displacements in the middle of the beam span were reduced by 12%. In the case of laboratory tests in beams reinforced with CFRP tape, the strain at points T1 and T2 was reduced by a maximum of 9.1 and 18.3%, respectively, and there was a decrease in vertical displacement by 15.4%. Therefore, the limitation of displacements and strain in the case of the laboratory tests was slightly greater than in the case of the numerical analyses.

Differences in displacement and strain results for numerical models with different lengths of CFRP tape were negligible and did not exceed 0.8%.

**Figure 9:**
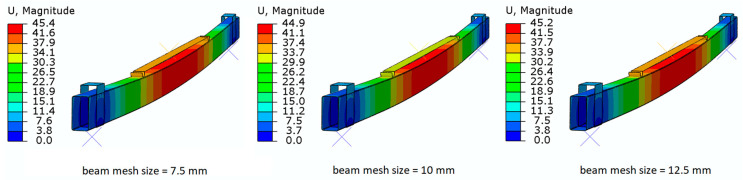
Effect of mesh density on FEA simulation results.

In addition, the effect of mesh density on the results of numerical calculations was verified within the framework of FEM simulations (as shown in Figure 9 in the example of the comparison of displacement levels). As a result, the selected mesh size of 10 mm in the case of the beam was the optimal mesh, and the results for this mesh were the closest in relation to experimental studies, both in terms of deformations, loads at which rupture of the composite tapes was registered, etc. A mesh with a lower density, e.g., 7.5 mm, and a higher density, e.g., 12.5 mm, showed larger deviations.

## 6. Comparison of Laboratory and Numerical Test Results

As can be seen, the use of CFRP tapes definitely contributes to the reduction in the displacements and strain of the beams subjected to the tests. At the same time, based on the conducted tests, it can be concluded that in the case of the deformation and displacement results, the impact of changing the tape length is minor, both in the case of the numerical analyses and laboratory tests. However, the anchoring length has a significant impact on the moment of the detachment of the CFRP tape and, thus, on the moment when the beam ceases to be reinforced.

**Figure 10:**
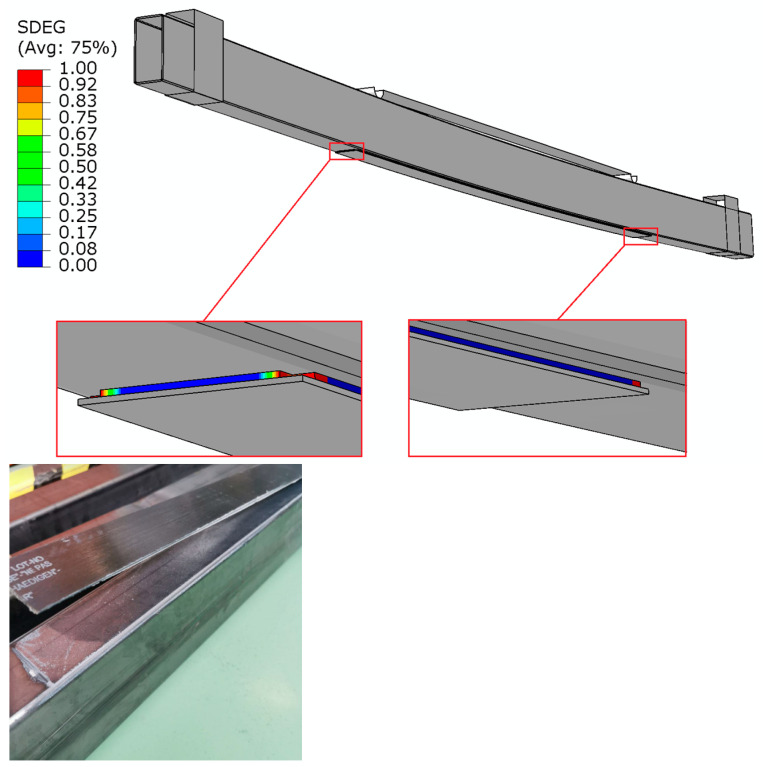
The damage of the glued joint during numerical analyses and laboratory tests.

During the laboratory tests, the value of the load was recorded at which the complete detachment of the composite tape from the surface of the steel beam took place. For some of the beams, detachment occurred along the entire length of the tape, while for others, it occurred on one half only. During numerical analyses, the load was recorded at the moment of the first damage to the adhesive layer. The load values are presented in Table 4. The damage of the glued joint during laboratory tests and numerical analyses is shown in Figure 10.

**Figure 11:**
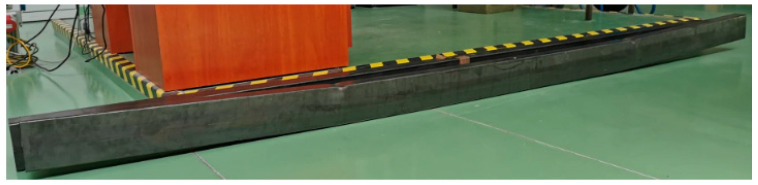
The failure of steel beams reinforced with CFRP tape with an anchorage length of 30 cm.

As can be noted, the load values obtained from the laboratory experiments and numerical analyses presented in the table differ, which results from the fact that the moment of the first damage to the adhesive layer was postponed relative to the moment of the detachment of the entire tape (the load value recorded in numerical analyses at which the first damage in the adhesive layer occurred is 8–12% lower than the load value level at which the tape was completely detached). The increase in the anchorage length from 7 to 15 cm slightly affected the increase in the load value at which the tape detachment took place. However, with an anchorage length of 30 cm, both tests show a very significant increase in the load at which the first damage occurred at the bonded joint. In the laboratory tests, the beams in which the anchorage length of 30 cm was used were yielding before the tape was detached (Figure 11), which is why this length seems to be the most appropriate anchorage length in the tested steel thin-walled beams.

## 7. Conclusions/Concluding Remarks

Based on the research carried out, it was found that:

- The reinforcement of a steel beam made of a profile with a rectangular cross-section with CFRP tapes with the smallest analyzed anchorage length contributes to a significant reduction in deformations in the compression (6.3%) and tension (16.5%) zones and a decrease in the value of vertical deflection in the middle of the beam span (9.3%) in relation to beams without reinforcement, with the quoted values referring to the load value level of 30 kN;
- An increase in the anchorage length from 7 to 30 cm in the tested beams resulted in a decrease in the value of strains in the compression flange by 2.8% (i.e., by 9.1% compared to reference beams) and a decrease in vertical displacements by 6.1% (i.e., by 15.4% compared to the reference beams);
- Analyzing the results obtained from the numerical analyses, the reinforcement in the form of CFRP tapes allowed the strain at points T1 and T2 in relation to the beam without reinforcement to be reduced by 8 and 17%, respectively, and the vertical displacements in the middle of the beam span to be reduced by 12%. Differences in the displacement and strain results for the numerical models with different CFRP tape lengths were negligible and did not exceed 0.8%;
- With the increase in the effective anchoring length, the value level of the load at which the tape detached at the adhesive–steel interface significantly increases; in the laboratory tests, increasing the effective length of the anchorage from 7 to 30 cm allowed a load level to be achieved at which beam yielding occurred before the tape detachment (an increase in the value of the load causing the tape detachment from 30.8 kN to no less than 41.95 kN). In the numerical analyses, the use of an anchorage length of 30 cm allowed a load level to be achieved at the moment of the first damage to the adhesive layer by 8.2 kN higher compared to the model with an anchorage length of 7 cm.

Taking into account the preliminary laboratory tests described in this paper, as well as the numerical analyses presented above, it can be concluded that in order to effectively strengthen thin-walled steel beams, it is most advantageous to use an effective anchorage length of 30 cm when using CFRP composite tapes.

## Figures and Tables

**Table 1:** The tested samples.

Sample	Length of the CFRP Tape (L_CFRP_)	Anchoring Length (L_Z_)
B1R, B2R	-	-
B1_7, B2_7	155 cm	7 cm
B1_10, B2_10	161 cm	10 cm
B1_15, B2_15	171 cm	15 cm
B1_30, B2_30	201 cm	30 cm

**Table 2:** Basic strength parameters of CFRP tapes and adhesive.

CFRP Sika CarboDur S512 Tape	
Poisson’s ratio	ν = 0.308
Young’s modulus	E = 165 GPa
SikaDur-30 adhesive	
Minimum compressive strength after 7 days	75 MPa
Compressive modulus	9600 MPa
Minimum tensile strength after 7 days	26 MPa
Minimum shear strength	16 MPa
Minimum peel strength after 7 days	21 MPa
Shrinkage	0.04%

**Table 3:** The values of strains and displacements of individual beams for the load level of 30 kN.

Tested Beam	Values Obtained for Each of the Tested Beams			The Arithmetic Mean for a Given Group of Results		
	T1	T2	LVDT2	T1	T2	LVDT2
	(×10^−6^)	(×10^−6^)	(mm)	(×10^−6^)	(×10^−6^)	(mm)
B1_7	−1906	1638	27.1	−1911	1588	27.0
B2_7	−1915	1538	27.0			
B1_10	−1868	1563	26.2	−1856	1563	26.4
B2_10	−1844	-	26.6			
B1_15	−1856	1575	26.6	−1864	1554	26.4
B2_15	−1873	1533	26.2			
B1_30	−1863	1608	25.7	−1853	1608	25.2
B2_30	−1842	1608	24.7			
B1_R	−2044	1899	30.0	−2040	1902	29.8
B2_R	−2037	1905	29.7			

**Table 4:** Load values corresponding to damage occurring within the glued joint.

Tested Beam	The Load Value at which the CFRP Tape Detachment Occurred—Laboratory Tests (kN)	The Arithmetic Mean of the Load Value at which the CFRP Tape Detachment Occurred—Laboratory Tests (kN)	The Load Value at which the CFRP Tape Detachment Occurred—Numerical Analyses (kN)
B1_7	30.4	30.8	28.6
B2_7	31.2		
B1_10	31.3	31.45	29.14
B2_10	31.6		
B1_15	31.3	31.75	30.35
B2_15	32.2		
B1_30	41.9—beam yielding before glued joint damage	41.95	36.8
B2_30	42.0—beam yielding before glued joint damage		

## Data Availability

Data is contained within the article.
